# Biosensors for Inorganic and Organic Arsenicals

**DOI:** 10.3390/bios4040494

**Published:** 2014-11-25

**Authors:** Jian Chen, Barry P. Rosen

**Affiliations:** Department of Cellular Biology and Pharmacology, Herbert Wertheim College of Medicine, Florida International University, Miami, FL 33199, USA; E-Mail: jianchen@fiu.edu

**Keywords:** arsenite, monosodium methylarsenate (MSMA) herbicide, methylarsenite (MAs(III)), roxarsone (Rox(V)), reduced roxarsone (Rox(III))

## Abstract

Arsenic is a natural environmental contaminant to which humans are routinely exposed and is strongly associated with human health problems, including cancer, cardiovascular and neurological diseases. To date, a number of biosensors for the detection of arsenic involving the coupling of biological engineering and electrochemical techniques has been developed. The properties of whole-cell bacterial or cell-free biosensors are summarized in the present review with emphasis on their sensitivity and selectivity. Their limitations and future challenges are highlighted.

## 1. Introduction

Arsenic is a toxic metalloid that is ubiquitously distributed throughout the Earth’s crust, soil, sediments, water, air and living organisms [1,2]. It is a Group 1 human carcinogen [3] and ranks first on the U.S. Comprehensive Environmental Response, Compensation and Liability Act (CERCLA) and Environmental Protection Agency (EPA) Priority List of Hazardous Substances. Arsenic enters the biosphere from both geological and anthropogenic sources. The adverse health effects of arsenic depend strongly on the dose, species and duration of exposure. Acute effects (short-term) of high-level exposure to arsenic range from gastrointestinal distress (nausea, diarrhea, abdominal pain) to death. Chronic (long-term) exposure to arsenic is associated with irritation of the skin and mucous membranes, cancer, neurological and cardiovascular events [4,5,6,7]. Environmental arsenic occurs mainly as inorganic species, and association with minerals or organic substances in soils limits its mobility. Considerable effort has been devoted to the detection of arsenic species and the mitigation of toxicity [8]. Due to its ubiquity and toxicity, the development of better methods for the detection of arsenic in water, soil and food is essential.

The maximum permissible concentration of arsenic in drinking water recommended by both the EPA and World Health Organization (WHO) is 10 µg/L [9]. Accurate measurement of such low levels of arsenic in drinking water requires expensive and sophisticated instrumentation and facilities, as well as trained staff. These techniques include atomic absorption spectroscopy (AAS), atomic fluorescence spectrometry, inductively-coupled plasma mass spectrometry (ICP-MS) and high-performance liquid chromatography with tandem mass spectrometroscopy (LC/MS/MS). Such techniques provide limits of detection well below the WHO/EPA arsenic guidelines, but are laboratory based, slow and expensive and not suitable for field testing [10,11,12]. Facile, rapid and sensitive methods for quantifying total arsenic and its species in aqueous samples are needed. Several recent reviews summarize the aspects of the field. Ma *et al.* discussed the progress in non-atomic spectrometric methods on speciation and detection of arsenic in aqueous samples and emphasized methods of analysis and speciation using spectroscopy and electrochemistry, ICP-MS, neutron activation analysis and biosensors [13]. A recent review describing analytical methods for sample treatment and speciation emphasizes that high performance liquid chromatography (HPLC) ICP-MS and hydride generation AAS are the most powerful methodologies for arsenic speciation in environmental and biological matrices [14]. These methods are reliable and can be used for the measurement of extremely low concentrations of arsenic. However, they suffer from major disadvantages, such as heavy and expensive instrumentation, lack of field applicability, the requirement for highly skilled technicians, sample processing and more. They are also time-consuming and not suitable for routine monitoring of large numbers of samples [15]. Alternative methods have been developed based on biological systems (e.g., bioreporters and biosensors) for the real-time detection of arsenic with cost- and time-efficient technologies. A number of biosensors have been developed in the last few decades. Biosensors have the advantages of sensitivity, specificity, simplicity, low manufacturing cost, low detection limit, fast response time, ease of use, portability and ability to furnish continuous real-time signals [16]. The aim of this present review is to provide an overview of the general mechanism of a biosensor and various types of arsenic biosensors developed in the past decades. The limitations with relevance to an arsenic biosensor and its new challenges in the future are discussed in this review. Numerous reviews described the advantages of the biosensor for the detection of arsenic in the environment, but very few address the limitations. In this review, we will highlight the challenges in biosensor development, such as detection specificity, detection multiplexing, keeping the biosensor alive and active for prolonged periods, and so on. This review will focus on the future development trends in the use of a general biosensor for arsenic detection in the environment.

## 2. The Development of Biosensors

### 2.1. Protein or DNA Based Biosensors

Sensors using nucleic acids for the recognition and monitoring of toxic compounds are useful because many toxins, such as arsenic, have high affinity for nucleic acids or for DNA binding proteins, and specific sequences can be detected rapidly and at low cost [17,18,19,20]. An electrochemical DNA-based biosensor for the detection of arsenic trioxide (As_2_O_3_) has been reported [21]. It should be pointed out that arsenic trioxide forms inorganic As(OH)_3_ (As(III)) when dissolved in solution at neutral pH. A voltammetric signal that reflects guanine oxidation decreases with exposure time and the concentration of As_2_O_3_, presumably as a result of a reaction between guanine and As_2_O_3_ that damages purine bases. The interaction of As_2_O_3_ with double-stranded DNA (dsDNA), single-stranded DNA (ssDNA) and 17-mer short oligonucleotide was observed electrochemically using differential pulse voltammetry (DPV) with a carbon paste electrode (CPE) at the surface and in solution [21]. Potentiometric stripping analysis (PSA) was employed to monitor the interaction of As_2_O_3_ with dsDNA in the solution phase using a renewable pencil graphite electrode (PGE). Changes in experimental parameters, such as the concentration of As_2_O_3_ and the accumulation time of As_2_O_3_, were assayed using DPV. However, the carbon paste electrode (CPE)-based DNA biosensor has limited sensitivity (detection limit of 1 mg/L) and cannot be used under harsh experimental conditions. An improved DNA biosensor with enhanced sensitivity was created using a Co(III) complex with 1,10-phenanthroline, [Co(phen)_3_]^3+^, as an electrochemical DNA marker, and the Ru(II) complex with bipyridine, [Ru(bipy)_3_]^2+^, as a DNA oxidation catalyst [22]. DNA, a target for oxidative damage by reactive oxygen species (ROS), was attached to the surface of a screen-printed carbon electrode. DNA damaged by arsenite, dimethylarsinic acid (DMAs(V)), phenylarsenate (PhAs(V)) and *p*-arsanilic acid (pASA(V)) was analyzed, and the DNA-based biosensor was higher for the aromatic arsenicals than for inorganic arsenic. However, there was no significant difference in the effect of individual organic arsenic compounds, so this sensor seems to lack selectivity. An advanced surface plasmon resonance-based DNA biosensor for As_2_O_3_ detection was developed using self-assembled monolayers for DNA immobilization [23,24]. The surface-sensitive analytical technique of surface plasmon resonance (SPR) was applied to monitor the binding of double-stranded calf thymus deoxyribonucleic acid (dsCT-DNA) with As_2_O_3_. The surface of the gold electrode was modified with 1-ethyl-3-(-3-dimethylaminopropyl) carbodiimide (EDC)/*N*-hydroxysuccinimide (NHS) on hydroxyl-terminated (OH) terminal self-assembled monolayers of β-mercaptoethanol (MCE). The study had a very low detection limit and a minimum response time, and the dsCT-DNA/2-ME/Au electrode is not selective for As_2_O_3_ and can only be utilized for the initial screening of drinking water and wastewater.

In addition to DNA-based biosensors, a variety of proteins have been used for sensing arsenicals. As(III) is a thiophilic metalloid that forms strong coordinate bonds with sulfur thiolates in proteins. Trivalent arsenicals inhibit or activate enzymes by binding to cysteine thiolates. Most protein-based biosensors developed for As(III) or As(V) are based on inhibition. An amperometric biosensor was developed to study the inhibition of acetylcholinesterase by As(III) [25]. The principle of the biosensor is based on the inhibitory effect of As(III) on the activity of acetylcholinesterase immobilized on a graphite electrode. In the presence of As(III), the levels of the thiocholine oxidation current were decreased proportionally to the As(III) concentration. The limitation is the action of As(III), as an acetylcholinesterase inhibitor is not specific. A number of other heavy metal ions, including Fe^3+^, Cd^2+^ and Cu^2+^, had a similar effect. Later, acid phosphatase (AcP)-polyphenol oxidase [26], arsenite oxidase [27], l-cysteine [28] and acid phosphatase [29] were each used for the construction of arsenic biosensors, but these also had limitations, such as a lack of specificity and low storage stability [30]. Recently, an electrochemical method based on a cytochrome c (Cyt c) biosensor was developed for the detection of arsenic [31]. Cyt c is a component of the electron transport chain in mitochondria and is sensitive to toxic compounds. Cyt c was immobilized on a boron-doped diamond electrode. Square-wave voltammetry (SWV) and electrochemical impedance spectroscopy (EIS) were performed to study the interaction of Cyt c with arsenic and cyanide. This biosensor can be prepared in a simple and quick manner, but it has a detection limit ranging from 1.6–4.3 mg/L for arsenicals that is much higher than the WHO/EPA limit of 0.01 mg/L.

### 2.2. Whole Cell-Based Biosensors

A whole cell-based biosensor is an analytical device that integrates whole cells, which provides high selectivity, with a physical transducer to generate a measurable signal proportional to the concentration of analytes [32]. Whole cell biosensors have the advantages of specificity, low cost, ease of use, portability and the ability to furnish continuous real-time signals [33]. The application of synthetic biology concepts to biosensor design highly improved the performance and broadened the range of application of these biosensors [34,35,36]. A whole-cell, man-made biosensor typically consists of a promoter, responsive to arsenic, genetically fused to a promoterless reporter gene. The recombinant genes can be located on plasmids or on the chromosome. An effective biosensor depends on the correct choice of its two constituents: the promoter and the reporter gene.

**Figure 1:**
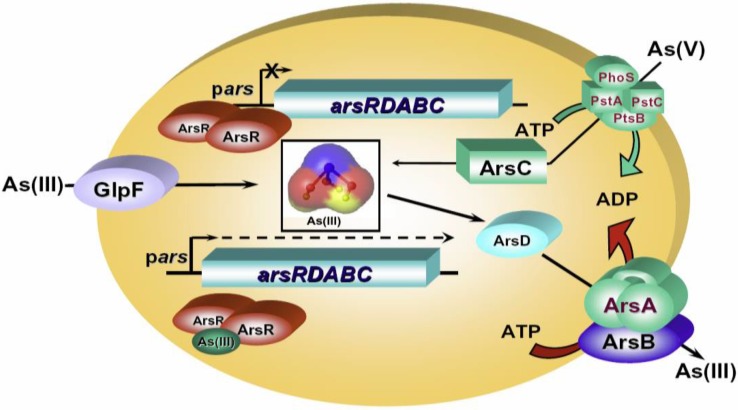
The *ars* operon of *E. coli* plasmid R773. Arsenic enters cells as either As(III) or As(V). As(III) is taken up by the aquaglyceroporin, GlpF, and As(V) is taken up by *pst* and *pho* phosphate permeases. The R773 *ars* operon has five genes, *arsRDABC.* ArsR is an As(III)-responsive transcriptional repressor that binds to the ars operator/promoter to prevent transcription in the absence of As(III). In its presence, ArsR binds As(III) and dissociates from the *ars* DNA, allowing the expression of the *ars* genes. ArsD is an As(III) chaperone that delivers it to the ArsA ATPase, the catalytic subunit of the ArsAB efflux pump. ArsC is a reductase that transforms As(V) to As(III).

The development of whole-cell living bacterial biosensors for arsenite was made possible by our discovery of the ArsR As(III)-responsive transcriptional repressor and its cognate promoter in 1990 [37]. The *ars* operon of *Escherichia coli* plasmid R773 consists of a set of structural genes (*arsA*, *arsB* and *arsC*) (Chen *et al.*, 1986) and two regulatory genes (*arsR* and *arsD*) [37,38]. *ArsR* controls the expression of other *ars* genes (Figure 1). ArsD is an arsenic metallochaperone transferring As(III) to ArsA ATPase [39]. ArsA and ArsB form an As(III)-translocating efflux pump, in which ArsA functions as the catalytic subunit and ArsB is the membrane subunit. ArsC is an arsenate reductase that converts arsenate to arsenite. In the absence of As(III), ArsR binds to its operator/promoter site within the *ars* operon and prevents further expression of itself and the downstream *ars* genes. When As(III) enters cells via an aquaglyceroporin [40,41], it binds to three cysteine residues in ArsR, leading to a conformational change, dissociation of ArsR from the operator DNA and subsequent expression of *ars* genes or the reporter gene, *lacZ* [42].

**Figure 2:**
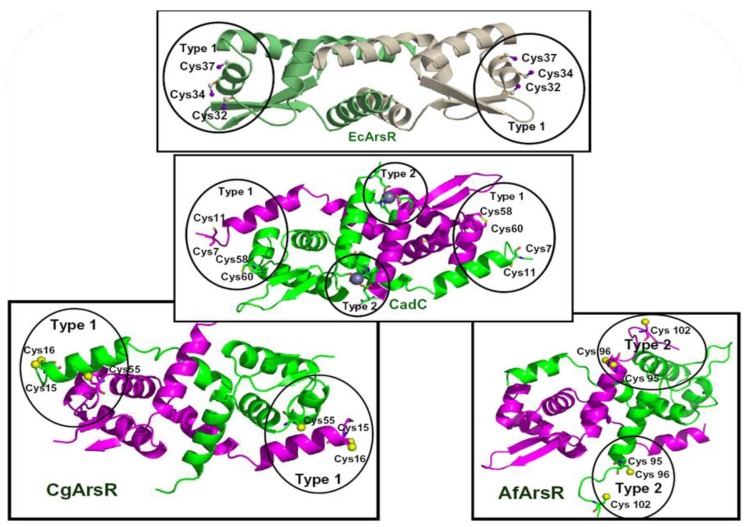
Structures of ArsR and CadC repressors. Homology models of three different ArsR repressors, EcArsR from *E. coli* plasmid R773, CgArsR from *C. glutamicum* and AfArsR from *A. ferrooxidans*, were constructed from the CadC crystal structure. The location and residues that comprise the Type 1 and Type 2 metal(loid) binding sites are indicated.

ArsR is the patriarch of a family of metal ion-regulated transcriptional repressors. Another member is the CadC Cd(II)/Pb(II)/Zn(II)-responsive repressor [43]. CadC binds its inducing metal ions at two cysteine residues in the DNA binding site [44,45]. The 1.9 Å X-ray crystal structure of the CadC homodimer has been valuable for understanding how arsenic is bound to ArsR (Figure 2). CadC has a Cd(II)/Pb(II)/Zn(II)-binding site composed of four cysteine resides, Cys7, Cys11, Cys58 and Cys60 (of which Cys11 is not absolutely required), in the putative DNA binding domain. CadC also has a second Zn(II) binding site that is structural and not regulatory. In addition to the R733 ArsR, two other ArsR orthologs have been identified with different As(III) binding sites. The As(III) binding site in the *E. coli* R773 EcArsR is composed of three cysteine residues, Cys32, Cys34 and Cys37 [42]. Modeling ArsR on the CadC structure allows visualization of the As(III) binding site in an N-terminal α helix that forms part of the DNA binding site. The difference between the two types of metal binding sites stems from the differences in four-coordinate Cd(III) and three-coordinate As(III), where the number and spatial orientation of the ligands contributes to metal ion selectivity. Moreover, the CadC structure and ArsR model suggest that binding of Cd(II) or As(III) results in a conformational change in the DNA binding domain, dissociation of the repressor from the operator/promoter DNA and derepression.

Two additional ArsR orthologs with different As(III) binding sites were identified in the *ars* operon of *Acidithiobacillus ferrooxidans* [46] and *Corynebacterium* *glutamicum* [47]. In contrast to the location of the As(III) binding site in EcArsR (which is termed a Type 1 site), the As(III) binding site in AfArsR (which is termed a Type 2 site), composed of Cys95, Cys96 and Cys102, is located at the *C*-terminus of the repressor (Figure 2). Homology modeling indicates that the two As(III) binding sites (one in each monomer) are located at the ends of antiparallel *C*-terminal α helices in each monomer that form a dimerization domain. Binding of As(III) can be predicted to unwind the helix and cause a conformational change in the dimerization domain that results in derepression. The As(III) binding site in CgArsR is yet again different. It is composed of Cys15, Cys16 from one subunit of the homodimer and Cys55 from the other subunit [48]. While it is located in a similar position to the EcArsR binding site, it is composed of different residues that form an inter-subunit binding site. Each ArsR has an As(III) binding site composed of three cysteine residues, because these form the strongest type of binding site for As(III). These results suggest that the As(III)-binding site in EcArsR, CgArsR and AfArsR each arose independently at spatially distinct locations in their three-dimensional structures.

Based on our demonstration that *arsR* could control the expression of reporter genes, a number of whole-cell-based biosensors containing an *arsR* gene plus the *arsR* operator/promoter fused to a reporter gene were developed. The marker gene encodes an easily measurable protein, such as luciferase, β-galactosidase or green fluorescent protein (GFP), whereby the signal relay is either in terms of luminescence, absorbance or fluorescence. Luciferase provides the most sensitive and simple measurement of gene expression and regulation. The first biosensors using luciferase (*luxAB*) in a fusion with the arsenic resistance operon used the expression of firefly luciferase controlled by the regulatory region of the *ars* operon of *Staphylococcus aureus* plasmid pI258 in recombinant plasmid pTOO21, with *S. aureus* RN4220, *Bacillus subtilis* BR151 or *E. coli* MC1061 as host strains [49]. Strain RN4220(pTOO21) was the most sensitive for metal detection responding to As(III), Sb(III) and Cd(II), the lowest detectable concentrations being 7.5, 2.5 and 25 µg/L, respectively. This illustrates two points. First, the choice of host strain can make a large difference in the efficiency of the biosensor. Second, Sb(III) is a much better inducer of the *ars* operon than As(III), because it is a stronger, more thiophilic metalloid. Following that study, we developed several biosensors using the R773 *arsR* gene in collaboration with S. Daunert. Expression of *lacZ* was monitored electrochemically using *p*-aminophenyl β-d-galactopyranoside as substrate [50]. This bacterial biosensor was very selective for As(III) and Sb(III) and showed no significant response to phosphate, sulfate, nitrate or carbonate. In a second biosensor, we used the bacterial luciferase genes, *luxA* and *luxB*, and showed that bioluminescence was proportional to As(III) and Sb(III) to 0.75 mg/L [51]. In addition, we used the gene for the ArsR homolog, CadC, to construct biosensors in which the expression of either *lacZ* or *gfp* was controlled by Cd(II) or Pb(II) [52].

The next step in biosensor development was to apply the concept to sensing of arsenic in soil. A luminescence-based bacterial sensor strain, *Pseudomonas fluorescens* OS8 (pTPT31), was developed in 2002 for arsenite detection in soil extracts [53]. The sensor strain with pTPT31 appeared to have a useful detection range similar to that of chemical methods. Moreover, unlike *E. coli*, *P. fluorescens* OS8 is a soil bacterium, which will be an advantage for future development of *in situ* bioavailability measurements under natural conditions. The biosensor was optimized by reducing reporter background expression in the absence of arsenic [54]. To reduce the background and increase the signal:noise, a second copy of the ArsR DNA binding site was placed between *arsR* and the respective reporter gene. This adjustment resulted in slightly lower background luciferase activity and reduced the response time by three- to five-fold at the same arsenite concentration. Three reporter proteins, bacterial luciferase, β-galactosidase and GFP, were tested in this biosensor. Field testing for arsenite was achieved with a system that contained β-galactosidase, producing a visible blue color at As(III) concentrations above 8 µg/L, which is in the range of the WHO/EPA recommended upper limit. The biosensor with luciferase as a reporter gene provided accurate and reproducible measurements at 4 µg/L. In fact, the biosensor was most quantitative with luciferase as the reporter, providing responses proportional within a concentration range of 8 to 80 µg of As/L. The sensitivity of the GFP biosensor to arsenite was less than that of the luciferase sensor, with a lower limit of detection of 8 µg/L. The concept has been commercialized with the development of field test kits, such as the ARSOlux Biosensor. An improved biosensor-based test kit comprised of *E. coli* DH5α–2697 harboring the *luxCDABE* genes under the control of *arsR* and its cognate promoter is available [55]. Compared to the testing rate of 60 samples per day with other commercial kits, this test kit achieved 160 samples per day. Recently, a biosensor system with a range of 0.7–60 μg As/L was reported that is both specific and selective for sensing bioavailable arsenic [56]. In this study, plasmid pASPW2 was the expression vector, and cyanobacterial *luxCDABE* expression was controlled by the *ars* promoter from a highly arsenic-resistant environmental isolate of *E. coli*.

GFP reporters have been developed in order to avoid the use of substrates that are needed for measuring the activities of reporters, such as β-galactosidase. GFP does not require the addition of a substrate or cofactor to produce fluorescence, and exposure to oxygen leads to spontaneous maturation of the fluorophore in the cell. Real-time assays are very useful for adaptation to high-throughput analysis, and GFP sensors have the potential for continuous, rather than end-point, measurements. An example is an arsenic-specific biosensor, *arsR* (and a small portion of *arsD*), and its upstream promoter fused with a green fluorescent protein from the marine jellyfish, *Aequorea victoria* [57]. When expressed in *E. coli*, this reporter creates a bacterial strain that produces GFP in response to arsenic exposure. GFP induction can be observed between 1 and 10 µg/L, but the response is not linear over the entire range tested, which was up to 10 mg/L. To improve the biosensor performance, a fluorescent microplate method was developed to detect arsenic using recombinant *E. coli* cells transformed with plasmids harboring three tandem copies of the report unit (*ars* operator/promoter *gfp* reporter gene fusion) [58]. This improvement lowered the detection limit to 7.5 µg/L, and the signal-to-noise ratio doubled without reducing the background noise. Microfluidic chips were developed using *E. coli* expressing *gfp* under the control of *arsR* with the long-term goal of the automation of dilution series and parallel detection of multiple analytes with integrated optics [59]. The fluorescent biosensor, *E. coli* DH5α pProbe-*gfp*(tagless)-*arsR*-ABS, was trapped on the biochip, which showed a linear response of the fluorescent signal as a function of exposure time and arsenite concentrations ≥50 µg/L. The drawbacks of this biosensor were a high detection limit and the requirement of a fluorescence microscope. A solid phase biosensor was developed in which the trans-cis element complex of the GFP-tagged trans factor was immobilized on the microwell surface, and dissociation of the complex was induced by binding of As(III) [60]. Water samples could be directly added to the complex, and arsenic could be quantified with fluorescence of the GFP-tagged trans factor released from the cis element. A detection limit of 5 μg/L for As(III) in purified water was achieved. A complex of lyophilized GFP-tagged ArsR bound to the cis element was stable at 4 °C. This system provides a suitable tool for on-site monitoring using a lyophilized solid phase, hand-held, portable fluorometer.

Recently, a compact portable biosensor for measurement of As(III) in water using *E. coli* strain 1598 has been developed. It carries the plasmid pPROBE-ArsR-ABS and produces GFP in response to As(III) and As(V) [61]. The biosensor was embedded in agarose mini-beads and placed on a microfluidics polydimethylsiloxane (PDMS) platform with two parallel channels for As(III) analysis. The biosensor exhibited highly reproducible measurements of arsenic in drinking water at 10 and 50 μg/L within 100 and 80 min, respectively.

The biosensors described above utilize prokaryotic reporter strains. A eukaryotic biosensor utilizing an arsenic-responsive fungal gene has been constructed [62]. *Aspergillus niger*, a plant pathogen used industrially for the production of citric and gluconic acids, has a number of arsenic tolerance genes. One, *acrA*, encodes an ortholog of the *Saccharomyces cerevisiae* Acr3 arsenite efflux permease [63,64]. The *acrA* gene is induced 200-fold by As(III), and an *acrA* promoter fusion with the *egfp* gene for enhanced green fluorescent protein can serve as a fluorescent biosensor. In *S. cerevisiae*, *acr1* is an As(III)-responsive transcription factor that controls the expression of *acr3*. *A. niger* does not have an *acr1* ortholog, so the basis for As(III) inducibility of *acrA* is not clear. An advantage of this system is that it appears to be more selective for As(III) or Sb(III) than the ArsR-based sensors. This fungal biosensor reliably detected both arsenite and arsenate in the range of 1.8–180 µg/L, exhibiting a low arsenic detection limit.

While biosensors have been developed for the measurement of inorganic arsenic, until recently, there have been no methods available for the detection of environmental organoarsenicals. Both methylated and aromatic arsenicals have been used for many decades for agriculture and animal husbandry. Pentavalent methylarsenate (MAs(V)) has been used as the herbicide monosodium methylarsenate (MSMA); approximately 3,000,000 pounds (1,360,000 kg) per year are in commercial use in the United States for the treatment of cotton fields, golf courses, turf management and highway medians. Until recently, it was sold in neighborhood lawn-and-garden stores as the Ortho product, Weed-B-Gone, for post-emergent grassy weed control in residential lawns. This herbicide is reduced by bacteria to more toxic and carcinogenic methylarsenite (MAs(III)) and to inorganic As(III) [65]. Pentavalent aromatic arsenicals, including roxarsone (Rox(V)), nitarsone (Nit(V) (4-nitrophenyl)arsonic acid) and *p*-ASA (*p*-aminophenyl arsonic acid), have been used since the 1940s as antimicrobial growth promoters for poultry and swine to control *Coccidioides* infections, improve weight gain, feed efficiency and meat pigmentation [66,67]. While Pfizer has voluntarily suspended production of roxarsone and pASA, they still make and sell Nit(V), which is the only known treatment for blackhead, or histomoniasis, in turkeys. In addition, roxarsone is produced world-wide and used in poultry farms in many countries. These aromatic arsenicals are largely excreted unchanged and introduced into the environment when chicken litter is applied to crops as fertilizer [66]. Pentavalent organoarsenicals are relatively benign and less toxic than inorganic arsenicals; however, both aromatic [66,67,68] and methyl [69,70] arsenicals are activated by reduction [71] and then degraded into more toxic inorganic forms in the environment, where they contaminate foods and water supplies. Diphenylchloroarsine (Clark I), which was used as a chemical warfare agent in World Wars I and II, is also degraded by microbial activity [2,6]. Monitoring the prevalence of these environmentally pervasive arsenicals requires methods for the detection of organic arsenic species in the field. For those reasons, we adapted our ArsR-based biosensors to detect organoarsenicals [71]. The objective of this study was to develop a biosensor that could specifically sense the reduced forms of MSMA (MAs(III)) and roxarsone (Rox(III)) without interference from inorganic arsenic. MAs(III) and Rox(III) are the active forms of the herbicide and antimicrobial growth promoter and are also obligatory intermediates in their breakdown, so the ability to sense the trivalent species is important to understanding their environmental impact. Current detection methods for total organic arsenicals in biological samples involve oxidative digestion of the organic matrix into inorganic arsenic, which is quantified by analytical laboratory techniques, such as ICP-MS. These laboratory-based spectroscopic methods are time-consuming, costly and require skillful operators. Commercial chemical field test kits are used in countries, such as Bangladesh and India, with varying degrees of success [72]. The principle of these kits is the formation of volatile arsine gas (AsH_3_) to separate arsenic from the aqueous matrix and subsequent colorimetric detection on a paper strip [73]. However, these test kits have low precision, poor reproducibility, high rates of false positives and negatives, and the accuracy is limited to concentrations between 10 and 100 µg/L [74]. Most critically, these methods cannot distinguish between inorganic and organic arsenic species.

**Figure 3:**
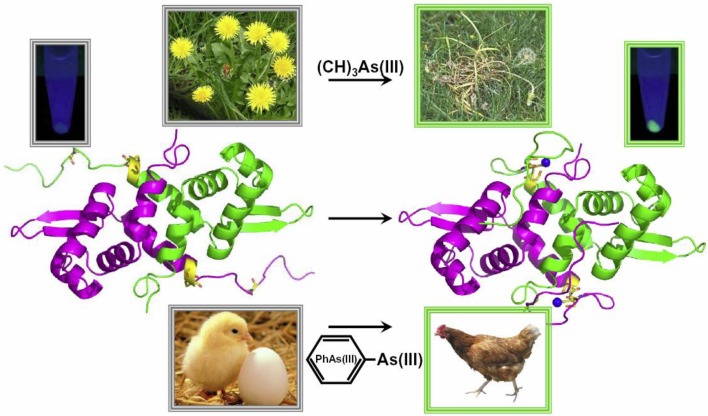
A biosensor for methylated and aromatic arsenicals. An *E. coli* cell-based biosensor was constructed that reports the sensing of environmental arsenicals by GFP fluorescence. The components of the biosensor are the gene for the AfArsR repressor, shown as the structural model, and the gene for GFP, shown by evolving fluorescence of the bacterial sensor in the minitubes. The biosensor responds to trivalent aromatic arsenicals, such as phenylarsenite (PhAs(III)) or Rox(III), with the highest affinity, responds to the reduced form (MAs(III)) of the herbicide MSMA with intermediate affinity and shows very low response to inorganic As(III).

**Figure 4:**
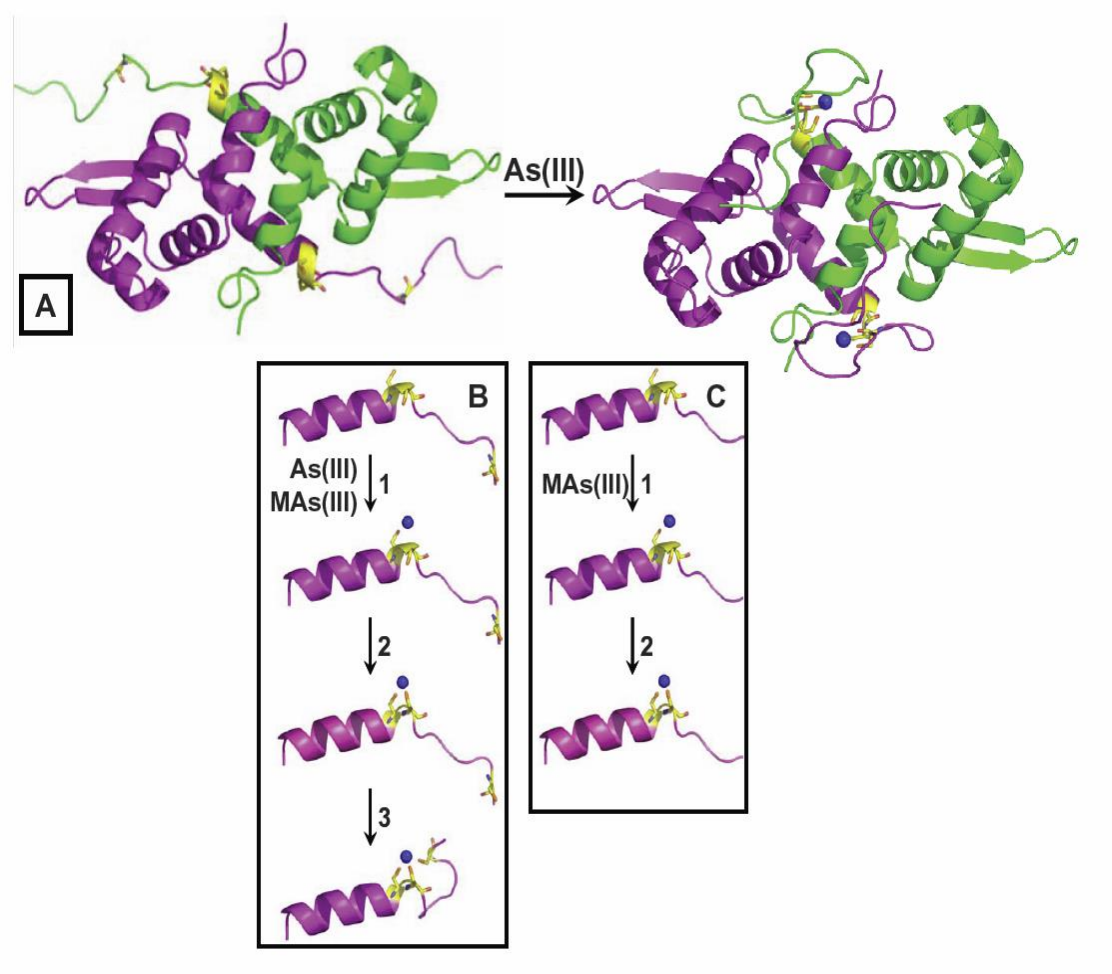
A genetically-engineered biosensor with selectivity for organoarsenicals. The *E. coli* cell-based biosensor was genetically modified to eliminate the As(III) binding site. The As(III) binding site in AfArsR is composed of three cysteine residues, Cys95, Cys96 and Cys102 (**A**). A C102S mutant loses the high-affinity binding of three-coordinate As(III), (**B**) but retains its affinity for two-coordinate organoarsenicals, such as MAs(III), PhAs(III) and Rox(III) (**C**).

To circumvent these problems, there is a novel biosensor selective for the active trivalent forms of the herbicide, MSMA, and roxarsone, as well as PhAs(III) and other aromatic arsenicals residues (Figure 3). A dual plasmid system was engineered in the arsenic-hypersensitive host strain, *E. coli* AW3110 [75], in which the first plasmid, pBAD*arsR*, had the *AfarsR* gene from *A*. *ferrooxidans* under control of the arabinose promoter, and the second plasmid, pACYC184-p*arsO-gfp*, had the *gfp* reporter gene from *Aequorea victoria* fused to the *A. ferrooxidans arsO* promoter [76]. By titrating the pBAD promoter with the inducer arabinose, the level of intracellular expression of AfArsR could be tuned, allowing the selectivity of the biosensor to shift from As(III) to PhAs(III) and MAs(III). While this biosensor was more selective for organoarsenicals, it still sensed inorganic As(III) at low levels. To improve selectivity, the *AfarsR* gene was engineered to eliminate the response to As(III) [71]. As described above, AfArsR uses three cysteine residues, Cys95, Cys96 and Cys102, to bind three-coordinate As(III). Reasoning that MAs(III), PhAs(III) or Rox(III) have only two available coordinations, we made a C102S derivative that could bind arsenicals to only two cysteine residues (Figure 4). This whole-cell biosensor is highly selective toward trivalent methyl and aromatic arsenicals, with essentially no response to inorganic arsenic.

## 3. Future Challenges

Exposure to arsenic is a global public health problem due to its ubiquity in the environment and its association with numerous human diseases. The development of biosensors for arsenic detection is of great value and interest not only to scientists, but to the public, as well. Construction and testing of new genetically-modified microorganisms for environmental sensing and reporting has proceeded at an ever increasing rate. During the last two decades, a number of arsenic biosensors have been developed based on both whole-cell- and cell-free-based biosensors. As discussed above, the performance of a number of biosensors could be improved. Their application has limitations in the detection limits, detection time and specificity. Most biosensors are still in a proof-of-principle or research phase and not close to commercial use. Pointing out the challenges of extant biosensors may assist in the development of realistic future objectives. Additional impediments to the development of practical devices for field studies are governmental regulations on the use of biosensors. This will undoubtedly vary from country to country. In the United States, the EPA regulates the use of transgenic organisms for commercial use as biosensors under the Toxic Substances Control Act (TSCA): “Microorganisms subject to this rule are ‘new’ microorganisms used commercially for ‘TSCA purposes,’ such as the production of industrial enzymes and other chemicals, agricultural practices (e.g., biofertilizers), biosensors, production of biofuels and the breakdown of chemical pollutants in the environment.” ‘New’ microorganisms are those in which genetic material from the organisms of different genera are added to the biosensor organism. Research and development studies are exempted, as noted in the EPA’s regulations of 2012. These may have changed or will change by the time this review appears, so readers are advised to contact the EPA for more recent regulations.

### 3.1. Challenges of Biosensor Specificity

Environmental arsenic is heterogeneous, with two different oxidation states of inorganic, methylated and aromatic species. Inorganic arsenic is pervasive in the environment from both geological and anthropogenic sources. Biological activities result in the incorporation of arsenic into organic molecules, such as arsenobetaine, arsenosugars and arsenolipids, which are found in many marine organisms [77]. Thus, it is imperative to be able to monitor both inorganic and organic arsenic contamination in the environment. However, most speciation and analysis methods are based on spectrometric and electrochemical methods, as well as inductively-coupled plasma-mass spectrometry. Most currently available bacterial whole-cell biosensors detect only inorganic arsenic. ArsR-based biosensors cannot distinguish between trivalent and pentavelant species. Even though ArsR is specific for As(III) and nonresponsive to As(V), most cells have arsenate reductases that transform As(V) to As(III). The exception are those based on *E. coli* strain AW3110, in which the *arsC* gene was deleted. Biosensor constructs were expressed in cells with and without an *arsC* gene, it might be possible to detect total arsenic (+*arsC*), As(III) specifically (−*arsC*) and As(V) from the difference between the two strains.

The whole-cell biosensor utilizing a genetically-engineered AfArsR repressor is highly selective toward trivalent methyl and aromatic arsenicals, because ArsC does not reduce aromatic arsenicals. However, commercial organoarsenicals are all pentavelant, even though their active forms are trivalent. That means that the majority of organoarsenicals are invisible to the biosensor strain. Bacteria species that reduce MSMA have been identified [65], so it should be possible in the future to clone the reductases and express them in the biosensor strains. Thus, the detection of the various environmental arsenic species is a critical challenge for biosensor development.

### 3.2. Challenges of Biosensor for Arsenic for In Situ Detection

Despite the development of promising arsenic biosensors, there are few studies that have critically evaluated arsenic bioassays under field conditions [78]. Most of the sensor assays discussed above have detection limits under laboratory conditions in the range of 10–50 µg/L, a desirable range, but this is often compromised by the presence of other ions or contaminants in water sample or field conditions that might complex arsenic or affect the viability of the bacterial biosensor cells. False positive signals are also possible if other substances elicit a positive response from the biosensors.

Another concern with arsenic biosensors is that they only respond to soluble arsenic, in contrast to total destructive chemical analyses. Improper sample preparation may significantly lower arsenic bioavailability to the cells, leading to underestimation of the total arsenic content of the sample [79]. Anoxic groundwater is often iron-rich, with concentrations in the range of 5–30 mg/L of iron, as well as varying concentrations of other ions, such as ammonia, bicarbonate, nitrate and silicate [80,81]. These can interact with arsenic to lower its mobility and detection. During groundwater sampling procedures, acids or complexing agents are usually added to preserve sample composition and prevent precipitation of arsenic onto iron(hydr)oxides (FeOOH) particles, which are rapidly formed when anoxic groundwater is exposed to air [82,83]. Thus, *in situ* environmental sample treatment is important for arsenic detection by biosensors.

On the other hand, biosensors have noteworthy advantages. Biosensors can be easily produced at low cost; biosensor assays are fast and easy to perform; the assays have sensitivity that typically meets or exceeds required standards; they report chemical bioavailability, rather than mere total concentrations. Yet, most biosensors have not been extensively field tested, and most are unlikely to reach commercialization stages. Thus, it remains a major challenge to develop biosensors for environmental applications.

### 3.3. Long-Term Challenges

Bacterial signal-responsive regulatory units have been employed as a platform to design and construct whole-cell bacterial biosensors for reporting toxicity. With the increased development in synthetic biology and understanding of arsenic-binding mechanisms, novel biosensors have been developed for arsenic determination [34]. However, little research has been conducted to address the largely unresolved concern of the “shelf-life” of the biosensor. It is essential that the cells have sufficient energy for sensing function within the formulated product. A weaker response will be obtained from cells with a compromised energy status. It is important for environmental biosensors to enable long storage at room temperature. Failure to maintain the survival and activity of biosensors will hamper their implementation. To meet these demands, various conservation techniques have been reported, including freeze drying, vacuum drying, continuous cultivation and immobilization in biocompatible polymers of organic or inorganic origin [84,85,86]. Different species and strains of bacteria exhibit great variability with respect to different preservation methods. Furthermore, the physiological status of biosensor cells upon dehydration is of importance in relation to their viability and activity after rehydration. It is important to construct a biosensor bacterium on the basis of its ability to be successfully formulated, rather than inventing new complicated sensing schemes using delicate microorganisms. For example, a biosensor based on naturally hardy *Bacillus cereus* spores has been reported to have excellent storage properties [87]. Thus, maintaining biosensor bacteria at ambient temperatures for prolonged periods is still a major challenge.

### 3.4. The Challenge of Multiplexing Biosensors

A multiplexed approach can be applied for the future development of biosensors. Biosensors can be combined with high-throughput, low-cost instrumentation for multiple analyses of environmental samples. For example, a pH-based biosensor has been developed for the detection of arsenic in drinking water [88]. This biosensor produces a pH-dependent color change in arsenic-contaminated samples. It allows sensitive and accurate detection of less than 10 µg/L arsenate with overnight incubation. It is a high-throughput system allowing continuous monitoring of up to 50 samples simultaneously. Another multiple-input biochip using a whole-cell based biosensor for arsenite detection couples biological engineering and electrochemical methods [89]. This whole-cell based biosensor couples the intracellular recognition of arsenite to the generation of an electrochemical signal. The arsenic-sensitive electrochemical biochip shows high sensitivity and selectivity. It has good sensitivity with tap water (0.8 µg/L) and can be achieved in a short time. High throughput detection of As(III) in drinking water can be monitored using this portable chip containing 16 independent two-electrode cells. Biosensors also are expected to be capable of simultaneously monitoring several environmental pollutants. Such biosensors may use a large number of recombinant bacteria, each monitoring the presence of a specific toxicant or genotoxic compound [90,91,92]. Multiplexing is a future challenge in the development of biosensors.

## 4. Conclusions

To date, a number of arsenic biosensors have been developed, some of which are based on whole-cell biosensors and others on cell-free (protein, DNA)-based biosensors. Some are reliable reporters of arsenic in environmental samples, including groundwater and soil. Considering their advantages—time and cost effectiveness, small size, robustness and ease of use—biosensors have the potential to be simple, sensitive and reliable solutions to the problem of monitoring arsenic in the groundwater of rural areas. At present, most biosensors are just laboratory curios, and their employ for environmental applications is limited. The require optimization of the detection limits, response times and specificity. The application of synthetic biology could take biosensors to new levels. For example, the introduction of signaling systems from other organisms could broaden the applicability of reporter assays for *in situ* environmental monitoring. Future challenges include the construction of biosensors for simultaneous monitoring of multiple environmental parameters.
